# The Change-Inventory for Career Counseling – An Instrument for Measuring Counseling-Correlated Changes

**DOI:** 10.3389/fpsyg.2020.01916

**Published:** 2020-08-07

**Authors:** Stephan Toggweiler, Hansjörg Künzli

**Affiliations:** School of Applied Psychology, Zurich University of Applied Sciences, Zurich, Switzerland

**Keywords:** career counseling, job counseling, career guidance, counseling-correlated changes, evaluation, VIL

## Abstract

Psychometric properties of a 23-item inventory that measures five correlates of career counseling for evaluation purposes are presented. The dimensions were developed bottom-up. The construction sample consisted of 3316 adult clients of public career counseling services in Switzerland, who were assessed within a naturalistic multicenter evaluation study with pre-post design. The inventory proved reliable (Cronbach’s α between 0.72 and 0.82, McDonnald’s ω between 0.73 and 0.81). Concerning validity, the dimensions were supported by exploratory and confirmatory factor analysis (CFI = 0.910, SRMR = 0.044, RMSEA = 0.056). Configural, metric and scalar measurement invariance of gender (female vs. male) and age group (<30 vs. ≥30 years) was also supported. Pre-post changes are medium to large. Practical use and theoretical localization among related German-language instruments are discussed.

## Introduction

### Occupational, Educational, and Career Guidance in Switzerland

Public occupational, educational and career guidance is defined within the Swiss Federal Law of Vocational Education and Training Act (article 49−51), which states that “occupational, educational and career counseling supports adolescents and adults in their occupational and academic choices and in designing their vocational career” (Zihlmann, 2009, p. 247). The main focus is on supporting successful transitions within vocational progressions. Furthermore, the qualifications of the counselors, the certification of courses and the responsibilities of the 26 cantons are noted. Occupational, educational and career guidance in Switzerland is usually linked to *vocational information centers*.

*Occupational guidance* supports adolescents in choosing an apprenticeship, *educational guidance* supports choosing a tertiary education or course of study, whereas *career guidance* helps adults choose a course of further study and/or plan their further career (usually, these activities take place in a 1:1 setting). During lower secondary level, youngsters attend *vocational preparation classes* for 2 years, where they are guided to think about their possible futures, to reflect on their interests and capabilities and, if necessary, to ask for support from guidance or vocational information centers. This system of *occupational*, *educational*, *career guidance* and *information centers* is provided in full by the cantons. The cantonal directors of occupational, educational and career guidance are members of the *Swiss Conference of the Directors of Occupational and Educational Guidance* (KBSB). This conference addresses all issues regarding coordination of occupational, educational and career guidance in the cantons. The *Swiss Service Center for Vocational Training*, *Study and Career Counseling* (SDBB) is run by the cantons, providing all kinds of information material for teachers, counselors and vocational information centers, hosting the agenda-setting commissions, as well as conducting quality assurance and trainings for the continuing education of experts in the field (Swisseducation, 2019).

Occupational, educational and career guidance systems in Germany and Austria are different to that of Switzerland (for a comprehensive overview see Euroguidance, 2019), but nevertheless concerned with the same issues and objectives as described below.

In Switzerland, career guidance usually takes place in the form of *career counseling.* This is a limited in time, goal-oriented process, in which, by means of a supportive 1:1 relationship and through conversations between counselor and client on a specific matter (aimed at helping clients take matters into their own hands), the coping strategies of clients are developed and sharpened (Zihlmann, 2009, p. 267; for Germany cf. also Rübner and Sprengard, 2010, p. 21). Regarding clients’ counseling concerns, they predominantly converge within these areas: choice of occupation or academic studies; search for an apprenticeship; problems with current vocational education and training (e.g., failing exams, performance problems, drop-out from apprenticeship); career design; occupational reorientation; occupational re-entry; unemployment or impending unemployment; financial issues; personal, social and health problems; serious problems in the workplace; integration issues of migrants (KBSB, 2016, p. 8). Depending on objectives and personal preferences, different methods and theoretical positions of counseling are used (cf. Patton and McIlveen, 2009; Rübner and Sprengard, 2010; Hirschi, 2013, 2015; Savickas, 2013; Nota et al., 2014b).

### Reasons for the Development of a New Instrument

However, the development of the *Veränderungsinventar für Laufbahnberatungen* (VIL) [*change-inventory for career counseling*], which is presented here, was preceded by the question of what constitutes successful counseling – which indicators, in the sense of core processes, must change positively in order to indicate successful counseling? Indeed, the development and application of outcome-measures is not new. Many Swiss studies measuring the effects of counseling have already been published (Hurni, 2007; Hirschi and Läge, 2008; Perdrix et al., 2010, 2012; Rossier et al., 2012; Hirschi et al., 2018) and from abroad (Crites and Savickas, 1996; Gottfredson, 1996; Germeijs and De Boeck, 2002; Brown et al., 2003; Perdrix et al., 2012; Savickas and Porfeli, 2012; Spurk and Volmer, 2013; Rübner et al., 2014; Rottinghaus et al., 2017; Schober and Langner, 2017). Furthermore, various meta-analyses examining the effectiveness of career counseling have been published (Spokane and Oliver, 1983; Oliver and Spokane, 1988; Whiston et al., 1998; Brown and Ryan Krane, 2000; Whiston et al., 2017). They conclude that counselings are usually effective, but unfortunately are mainly restricted to college student or school pupil samples and are conducted within laboratory designs or settings that have little to do with the needs of practitioners in the field. Thus, it seemed promising to initiate the bottom-up development of a new instrument that sought a direct reference to the daily practices of career counselors. With this target in mind, the Swiss Conference of the Directors of Occupational and Educational Guidance (KBSK) and the Zurich University of Applied Sciences (ZHAW) decided to develop a psychometrical evaluation tool called *Veränderungsinventar für Laufbahnberatungen* (VIL). This *change-inventory for career counseling* was explicitly constructed from and for practitioners counseling adults, but with the demands of psychological research in mind. It therefore had to be action-oriented and capable of mapping a counseling process with as few items as possible, but with reliable scales.

### Development of the Instrument

The preliminary development phase of the VIL, from 2003 to 2004, was dedicated to the bottom-up development of items: On the basis of several workshops with experienced career counselors from different cantons of Switzerland, ideas were collected and items were formulated concerning the experiential and behavioral aspects of adult clients that counselors regard as indicators of successful career counseling. The guiding question was: with what mindset are clients ideally expected to leave career counseling once it is finished. Furthermore, these changes should be apparent independently of any particular career and counseling theory or method.

The second phase of development (2005−2006) is documented in Künzli and Zihlmann (2008). It comprised the further development of the item contents as well as a field phase with psychometric analysis, in particular factor analyses. While formulating the items, Künzli and Zihlmann (2008) were at least partially guided by the Rubikon model (Heckhausen, 1987; Grawe, 2000; Heckhausen and Heckhausen, 2006) and related concepts referring to consulting processes. “Our new items are not strictly derived from these models, but they do formulate feelings, impressions and general appraisals which clients often express spontaneously in the problem-solving process and which can be linked to presumed motivational and volitional processes” (Künzli and Zihlmann, 2008, p. 115). This study was the first evaluation of real-life counseling sessions with the VIL, in which six regional public counseling centers as well as a private provider participated. A total of 116 counselors took part in the study, each contributing up to 40 clients. The average client age was 31 years (*SD* = 9.8), 51.4% were female, 38.4% male and 10.1% were missing as to gender (Künzli and Zihlmann, 2008, p. 116). The target group for the evaluation were all adult clients, regardless of their counseling concerns; however, if their counseling concern was the choice of a first occupation, they were not included in the sample.

In the third and last phase of development the content of the items was checked again and was in some cases slightly modified. Table 1 shows these changes. These were in particular changes relating to the Cronbach’s α, relating to redundancies of the contents or changes relating to precautions which appeared to be useful for maintaining the factorial simple-structure of the items. No new scales were added. Only highly selective changes were made, which closely adhered to the already existing factorial structure. *Informedness about the change processes* was as early as 2008 the least satisfactory scale in terms of psychometrics and hence was dropped; this was also because of low applicability to the counseling process and general test economy. If possible, its items were reallocated to other scales. The result was the current change-inventory for career counseling VIL, as reported here. Since the resulting VIL is still in regular use and regarded from practitioners as up-to-date concerning contents and practical implications, this instrument is published here.

**TABLE 1:** Changes from the original VIL (Künzli and Zihlmann, 2008) to the current VIL.

Scales and items from Künzli and Zihlmann (2008)	Cause of change	Scales and items definitively used here
Wohlbefinden (α = 0.84)		Wohlbefinden [well-being]
Momentan fühle ich mich sehr ausgeglichen.	−	Momentan fühle ich mich sehr ausgeglichen. [At the time being I feel very balanced.]
Zurzeit fühle ich mich recht angespannt.	−	Zurzeit fühle ich mich recht angespannt. [At the time being I feel pretty tense.]
Momentan fühle ich mich sehr wohl in meiner Haut.	−	Momentan fühle ich mich sehr wohl in meiner Haut. [At the time being I feel very comfortable.]
Ich fühle mich unter Druck.	Redundancy of content; inflated Cronbach’s α	−
Ich bin unzufrieden, ohne genau zu verstehen, warum.	Redundancy of content; inflated Cronbach’s α	−
Informiertheit (α = 0.71)	−	Informiertheit [information]
Ich kenne meine Aus- und Weiterbildungsmöglichkeiten.	−	Ich kenne meine Aus- und Weiterbildungsmöglichkeiten. [I know my education and training opportunities.]
Ich kenne mich mit all den Möglichkeiten (Berufen, Ausbildungen, etc.) zu wenig aus.	−	Ich kenne mich mit all den Möglichkeiten (Berufen, Ausbildungen, etc.) zu wenig aus. [I know too little about all the possibilities (professions, trainings, etc.).]
Ich verfüge über genügend Informationen zu den Möglichkeiten, die für mich in Frage kommen.	−	Ich verfüge über genügend klare Informationen zu den Möglichkeiten, die für mich in Frage kommen. [I have clear enough information about the possibilities that are appropriate for me.]
Ich weiss, wie ich zu wichtigen Informationen komme.	−	Ich weiss, wie ich zu wichtigen Informationen komme. [I know how to get key information.]
Ich weiss, was ich lernen und unternehmen muss, um meine Wünsche zu erfüllen.	Redundancy of content	−
-	Reassurance of Cronbach’s α	Ich bin über die beruflichen Möglichkeiten, die mich interessieren, gut informiert. [I’m well informed about the career opportunities that interest me.]
Vertrauen in Entwicklungsperspektiven (α = 0.83)	−	Vertrauen in Entwicklungsperspektiven [trust in future perspectives]
Ich sehe Möglichkeiten, in denen ich meine Interessen und Fähigkeiten verwirklichen kann.	−	Ich sehe Möglichkeiten, in denen ich meine Interessen und Fähigkeiten verwirklichen kann. [I can see opportunities in which I can apply my interests and abilities.]
Ich sehe (berufliche) Möglichkeiten, die zu mir passen.	−	Ich sehe (berufliche) Möglichkeiten, die zu mir passen. [I see (professional) opportunities that suit me.]
Ich glaube, dass ich auf dem richtigen Weg bin.	Easier interpretability of the item	Ich bin überzeugt, dass ich auf dem richtigen Weg bin. [I’m convinced that I’m on the right track.]
Ich sehe viel versprechende Spuren in meine Zukunft.	−	Ich sehe viel versprechende Spuren in meine Zukunft. [I see promising tracks into my future.]
Ich freue mich auf die anstehenden Veränderungen.	Inflated Cronbach’s α	−
Ich blicke zuversichtlich und heiter in meine Zukunft.	Inflated Cronbach’s α	−
Informiertheit über Veränderungsprozesse (α = 0.60) [informedness about change processes]	Scale was psychometrically unsatisfactory in 2008	−
Ich weiss, was für ein Umfeld ich brauche, damit ich meine Stärken am besten entfalten kann.	Avoid possible loading on scale *information*	Ich bin mir im Klaren, was für ein Umfeld ich brauche, damit ich meine Stärken am besten entfalten kann. [I am aware of what kind of environment I need so that I can best develop my strengths.]
Ich kann deutlich zwischen für mich passenden und nicht passenden Lösungen unterscheiden.	−	Ich kann deutlich zwischen für mich passenden und nicht passenden Lösungen unterscheiden. [I can clearly distinguish between solutions that are suitable for me and solutions that are not.]
Ich weiss, wie ich Veränderungen in Angriff nehmen muss.	Elimination of the scale *informedness about change processes*	−
Ich weiss, wie viel Aufwand eine Veränderung mit sich bringt.	Elimination of the scale *informedness about change processes*	−
Zielklarheit (α = 0.85)	−	Zielklarheit [goal clarity]
Momentan habe ich keine Ahnung, was ich will.	−	Momentan habe ich keine Ahnung, was ich will. [Right now, I have no idea of what I want.]
Ich kenne meine Ziele.	Avoid possible loading on scale *information*	Ich tappe bei beruflichen Zielen im Dunkeln. [My career goals are unclear.]
Ich habe konkrete Vorstellungen, was ich will.	−	Ich habe konkrete Vorstellungen, was ich will. [I have clear ideas of what I want.]
Ich habe momentan keine klaren Ziele.	Item is too polarizing	Ich habe nur unbestimmte Ideen, in welche Richtung es gehen könnte. [I only have vague ideas as to which direction I might move in.]
Ich habe Ideen für konkrete Berufsfelder, in denen ich tätig sein möchte.	Relates to the target group of occupational first choice	−
Mir ist klar, welches die nächsten Schritte sind, die ich in Angriff nehmen werde.	Avoid possible loading on scale *certainty*; inflated Cronbach’s α	−
Unsicherheit (α = 0.75)	Polarity reversal for the purpose of simpler interpretability	Sicherheit [certainty]
Ich frage mich, ob ich mit meinen Zielen richtig liege.	−	Ich frage mich, ob ich mit meinen Zielen richtig liege. [I wonder whether I’m heading in the right direction.]
Ich drehe mich bei der Suche nach (beruflichen) Lösungen im Kreis.	−	Ich drehe mich bei der Suche nach (beruflichen) Lösungen im Kreis. [I go around in circles searching for (professional) solutions.]
Ich zweifle daran, ob die Möglichkeiten, die mir durch den Kopf gehen, wirklich in Frage kommen.	−	Ich zweifle daran, ob die Möglichkeiten, die mir durch den Kopf gehen, wirklich in Frage kommen. [I doubt that the possibilities that I am thinking about are really possible for me.]
Ich bin verunsichert, was meine Zukunft anbelangt.	−	Ich bin verunsichert, was meine Zukunft anbelangt. [I’m confused about my future.]
Ich fühle mich unsicher, wie ich meine Zukunft gestalten will.	Redundancy of content	−
–	Additional aspect; reassurance of Cronbach’s α	Ich zögere, die Umsetzung eines Plans an die Hand zu nehmen. [I hesitate in implementing a plan.]

### Theoretical Location of the Scales

The VIL comprises the five scales *well-being*, *information*, *trust in future perspectives*, *goal clarity* and *certainty*. There are several instruments with related contents; these are shown in Table 2 and are referred to in the following theoretical explanations of the VIL scales.

**TABLE 2:** Comparative representation of the VIL with related instruments.

VIL	MVS (Holland et al., 1980; Hirschi and Herrmann, 2013)	CAAS (Savickas and Porfeli, 2012; Johnston et al., 2013)	CFI-R (Rottinghaus et al., 2012; Rottinghaus et al., 2017; Spurk and Volmer, 2013)	CRQ (Hirschi et al., 2018)
Well-being			Work-life balance, Negative career outlook	Job challenge
Information	Occupational information	Curiosity	Occupational awareness	Job market knowledge, Career exploration
Trust in future perspectives	Barriers	Confidence	Support	Occupational expertise, Soft skills, Career opportunities, Organizational career support, Social career support, Career confidence, Learning
Goal clarity	Identity	Concern	Career agency	Career involvement, Career clarity, Networking
Certainty	Decision-making	Control		
German	German/English	German/English	English	German/English

#### Well-Being

On the one hand, *well-being* refers to life satisfaction; on the other, it refers to the prerequisites for a fulfilling life and human strengths. People experience psychological well-being, “if they can act autonomously in their lives, master environmental demands, experience personal growth, cultivate positive relationships with other people, recognize meaning in life and accept themselves” (Wirtz and Strohmer, 2013, p. 1677). Knowing a client’s level of *well-being* is important for each and every counselor. Unresolved developmental tasks, missing perspectives and loss of control can place people under negative tension and stress (Bischof, 1997, p. 325). It is possible that there are insufficient resources available to cope with such demands, which means that an individual finds himself in an increasingly stressful situation. One of the aims of successful coping (here: career planning) is to restore the client’s level of *well-being* by means of an appropriate problem-solving process (Wirtz and Strohmer, 2013, p. 340).

The *Career Futures Inventory* (CFI-R) of Rottinghaus et al. (2017) also uses measures of *well-being* via the scales *work-life balance* (e.g., “I can easily manage my needs and those of other important people in my life”) *and negative career outlook* (e.g., “Thinking about my career frustrates me”). In Hirschi et al.(2018, p. 347), a connection with career satisfaction and self-realization is established in their recently published *Career Resources Questionnaire* (CRQ) on the basis of the scale *job challenge* (e.g., “My work allows me to fully utilize my professional skills.”) – in this case, the absence of *job challenge* would suggest dissatisfaction and reduced *well-being*. Also, the widespread *Career Construction Theory* of Savickas (2012, 2013) and its very well studied *Career Adaptability Scales* (CAAS; Savickas, 1997; Savickas and Porfeli, 2012) containing *concern*, *control*, *curiosity* and *confidence*, each of them a relevant predictor of coping with occupational development, transitions and traumas (cf. Savickas and Porfeli, 2012, p. 661), are generally seen as being closely connected with *stress experience*, *occupational self-efficacy* and *self-esteem* (Johnston et al., 2013, p. 296; Savickas and Porfeli, 2012, p. 662). There is also clear proximity to the *Satisfaction with Life Scale* of Diener et al. (1985), to *temporal life satisfaction* (Trautwein, 2004), to *career satisfaction* (Greenhaus et al., 1990) and to the *comfort* scale of the *Career Decision Profile* (Jones, 1989, p. 479), where it is used as an indicator for an appropriate or inappropriate decision-making process.

#### Information

Besides intrapsychological coping, action execution and action omission, the availability and search for information is one of four basic coping strategies (Wirtz and Strohmer, 2013, p. 341). *Information* (in the sense of information about possibilities) “enables clients to get an overview and to imagine the future more precisely. Information is therefore a means of familiarizing oneself with target areas (occupational fields, particular occupations, occupational trainings, occupational functions, interim solutions, etc.).” (Künzli and Zihlmann, 2008, p. 121). The provision and acquisition of information is (besides counseling and support in implementation) a core task of career counseling (Zihlmann, 2009, pp. 259ff.). Item example: “I know too little about all the possibilities (professions, trainings, etc.).”

An *occupational information* scale has already been integrated into *My Vocational Situation* (MVS) of Holland et al. (1980). As part of the *Career Adaptability Scales*, Savickas(1997, p. 667; Savickas and Porfeli, 2012) also takes up the topic of being informed as *curiosity* (e.g., “Investigating options before making a choice”). Hirschi et al.(2018, pp. 346ff.) integrated the scales *job market knowledge* (e.g., “I have a good knowledge of the job market”) and *career exploration* (e.g., “I regularly collect information about career opportunities”). Rottinghaus et al. (2017); cf. also (Spurk and Volmer, 2013) also have their *occupational awareness* (e.g., “I keep current with job market trends”) in close proximity with the VIL *information* scale. Furthermore, *information* can be considered as being in close proximity to the *lack of information* scale of the *Career Decision-Making Difficulties Questionnaire* (Gati et al., 1996) or to the *knowledge about occupations and training* scale of the *Career Decision Profiles* (Jones, 1989).

#### Trust in Future Perspectives

The *trust in future perspectives* scale comprises “the premonition of having found a path suitable for one’s development […]. It is the belief in being on the right track regarding the challenges of the future, even though they are but vaguely known.” (Künzli and Zihlmann, 2008, p. 122). Item example: “I can see opportunities in which I can apply my interests and abilities.”

In Holland et al. (1980) *My Vocational Situation*, this aspect is particularly reflected in the *perceived barriers to career development* scale, although as an opposite. This scale includes, for example, too high training costs, difficulties in finding a job, restrictions due to family commitments, fears concerning important reference persons, or other aggravating aspects (Holland et al., 1980; Joerin Fux et al., 2013, p. 21). *Trust in future perspectives* is explicitly taken into account within the *Career Adaptability Scales* (Savickas and Porfeli, 2012, p. 667) in the form of the *confidence* scale (e.g., “Overcoming obstacles”), and in Rottinghaus et al.(2017, p. 69) in the form of the *support* scale (e.g., “Others in my life are very supportive of my career”). Hirschi et al. (2018) represent this aspect in a number of scales, e.g., the *occupational expertise* scale (e.g., “Others see me as an expert in my occupation,” measuring the confidence in one’s own abilities and value to the market), *soft skills* (e.g., “I have many skills that I could use in a range of different occupations”), *career opportunities* (e.g., “My organization holds many interesting positions for my future career”), *organizational career support* (e.g., “I feel fully supported in my career development by my current employer”), *social career support* (e.g., “I receive a high level of career support from my social environment”), *career confidence* (e.g., “When I set goals for my career, I am confident that I can achieve them”), and *learning* (e.g., “I make sure that my work-related abilities and knowledge are up-to-date”). Savickas (2005) relates the *confidence* scale into close proximity to self-esteem and self-efficacy (Johnston et al., 2013, p. 296), as both are indicators of trust in one’s abilities and skills.

#### Goal Clarity

An essential task of counseling is to anticipate different vocational outcomes on the basis of the available information, to clarify goals and to define them conclusively. Ultimately, *goal clarity* is a necessary prerequisite to monitor planned behavior, for both the maintenance of a specific vocational target and the attainment of a specific *status quo* (cf. Flammer, 1990, p. 78). The *goal clarity* scale measures the existence of such target areas and planned behavior goals. The availability of vivid vocational goals is also responsible for endurance and frustration tolerance (Heckhausen and Heckhausen, 2010; Höge et al., 2012; on personal goals and life plans see Brandtstädter and Lindenberger, 2007, pp. 270ff.). Example: “I have clear ideas of what I want.”

Concerning content, the *goal clarity* scale is related to the *identity* scale of the *My Vocational Situation* (Holland et al., 1980; cf. also Joerin Fux et al., 2013, p. 21), which represents the knowledge of one’s own goals, interests, talents and personality. Within the *Career Resources Questionnaire* of Hirschi et al.(2018, pp. 342ff.) *goal clarity* is somewhat similar to the scales *career involvement* (e.g., “I feel strongly attached to my work”), *career clarity* (e.g., “I have a clear understanding of what I want to achieve in my career”) and *networking* (e.g., “I always try to be well connected in my aspired professional field”). In the case of Savickas and Porfeli (2012), the *concern* scale (e.g., “Planning how to achieve my goals”) is likely to have the greatest similarity with *goal clarity*.

#### Certainty

*Certainty* means a state of decisiveness regarding one or more vocational options. It is a matter of deciding on a current goal from a large number of possible goals and a matter of maintaining this goal as decisive for upcoming actions (Flammer, 1990, p. 78). The degree of this decisiveness and conviction is measured by the *certainty* scale. If the result of this evaluation is positive, the prerequisites for transferring the counseling results are advantageous – otherwise one remains ambivalent or may even abandons a chosen goal. In the counseling situation, this scale can be addressed, for example, by applying the motivation principle of maximizing the expected benefit. The motivation principle states that “out of several possible alternatives, the one with the highest expected value is chosen” (Wirtz and Strohmer, 2013, p. 491). Example: “I wonder whether I’m heading in the right direction.”

According to Holland’s operationalization of the *vocational identity* subscale within *My Vocational Situation* (Holland et al., 1980; cf. also Joerin Fux et al., 2013, p. 21), *vocational identity* means, among other things, the ability to make convincing decisions, which is represented in the subscale *decision-making* (e.g., “I am not sure that my present occupational choice or job is right for me.”). Regarding this aspect, there is a particularly large number of highly specialized instruments (e.g., Osipow, 1987; Jones, 1989; Betz et al., 1996; Gati et al., 1996; Germeijs and De Boeck, 2002; Betz et al., 2005). Within the *Career Adaptability Scales* (Savickas and Porfeli, 2012), *certainty* finds its counterpart in the *control* scale (e.g., “Doing what is right for me”).

### Scope of the VIL

In short, one may say that the scales of the VIL have a number of similarities to other instruments with similar intentions. Thus, the scales of the VIL (theoretical considerations suggest that; empirical results do not yet exist) address all areas of the *My Vocational Situation* (Holland et al., 1980; Hirschi and Herrmann, 2013), as well as the dimensions of the *Career Adaptability Scales* (Savickas and Porfeli, 2012; Johnston et al., 2013), on which first intervention programs are already based (e.g., Nota et al., 2014a). Furthermore, the revised version of the *Career Futures Inventory* (Rottinghaus et al., 2017) is also covered by the VIL, as well as the *Career Resources Questionnaire* of Hirschi et al. (2018).

What makes the VIL unique compared to the instruments mentioned above is that the VIL was developed explicitly for evaluation purposes under real-life conditions and in direct cooperation with career counselors. This ensures that its scales represent “natural” intentions of career counseling interventions and – because of its independence from counseling theories and methods (the VIL does not aim to fit into any career model or theory, but aims to depict a counseling process including outcome) – are widely accepted in practice. By obtaining its frame of reference from practical experiences, the VIL is rooted therein. This enables the VIL to depict a process and target status that can be attained through the commonly practiced *status quo*. Correlations with the instruments mentioned above can now be tested empirically; first studies are in preparation, with the presumptions being listed in Table 2. Another uniqueness is that the VIL is closely located to the psychology of action, and this orientation toward action makes the VIL highly attractive for practitioners.

### Target Groups of the VIL

The change inventory VIL was designed for all adult career counseling clients, either youngsters starting from the second year of vocational education and training or attending schools of general education in the 10th grade or above (upper secondary specialized schools, other upper secondary schools, baccalaureate), as well as tertiary level students, academics and career counseling candidates with vocational qualifications. A characteristic feature of this target group is that it deals with career questions as they arise during or after general educational schooling or vocational education and training. Other constructs with evaluative purposes, typically based on the matching paradigm, are used for first-time occupational choosers, who are mainly concerned with questions of entry into post-compulsory education, i.e., occupational choice (e.g., Hirschi, 2007, 2008; Künzli and Toggweiler, 2014; Rübner et al., 2014; Jungo and Egloff, 2015). Regarding the reasons for visiting a vocational counselor, the VIL considers the following: career planning, vocational reorientation; choice of study; problems with vocational education and training (exams, performance, dropping out); occupational re-entry; unemployment, impending unemployment; personal, social and health problems; problems at the workspace; integration issues of migrants.

### Pre-post Effect Sizes

The pre-post effect sizes of the original VIL, reported by Künzli and Zihlmann(2008, p. 124) and in terms of Cohen’s *d* for differences in means of two equally large groups (cf. Table 4) are highly significant for each scale, whereby *goal clarity* (*d* = 1.22), *information* (*d* = 1.73), *informedness about change processes* (*d* = 0.90), and *uncertainty* (*d* = 1.28) revealed large effect sizes, *trust in future perspectives* (*d* = 0.65) and *well-being* (*d* = 0.70) medium effect sizes.

Women were overrepresented in the sample at 65.1%. The majority of respondents (86.2%) were more than 19 years old and had completed basic vocational education (33.3%). About 60% of clients indicated career planning or vocational reorientation as the reason for visiting the public center for occupational and career counseling. The duration of the counseling was skewed to the right with a median of 87.16 min and with a median of 1.45 sessions.

### Data Analysis

As a first step, items with negative polarity were reversed. For item and scale analyses (hypothesis 1), the distribution characteristics of the items and scales, the part–whole correlations, the internal consistencies according to Cronbach’s α and McDonnald’s ω were calculated. A cut–off value of 0.30 was assumed for the part–whole correlations and 0.70 for Cronbach’s α and McDonnald’s w. With these cut–off values the instrument would have sufficient measurement accuracy to be suitable for individual diagnostics (Nunnally and Bernstein, 1994, pp. 264f.; Schermelleh–Engel and Werner, 2012, pp. 135f.).

To examine the factorial structure of the VIL (hypothesis 2) an exploratory factor analysis (EFA) was conducted. The EFA was done with the Maximum Likelihood method followed by oblimin rotation. Furthermore, to gain hypotheses about a higher order structure (general and/or group factors), the extracted factor scores for each participant were untertaken further EFA’s, based on the Maximum Likelihood method followed by varimax rotation to get the group factors as independent as possible.

IBM SPSS Amos 26 was used to test the proposed 23–5 measurement model by confirmatory factor analysis (hypothesis 3) using the Maximum Likelihood (ML) method against the competing 23–5–1 model, as well as against other models derived within hypothesis 2. The ML method is recommended as standard by Bühner(2011, p. 413). The VIL scales deviated only slightly from a normal distribution and were all below the cut–off values defined by West et al.(1995, p. 74), that is, skewness ≤ —2— and kurtosis ≤ —7—. Moreover, the ML method is robust against violations of the normal distribution assumption, especially in the case of large samples (Marsh, 2007, pp. 780f.). The regression weights of all error terms were set to: (1) According to Marsh(2007, pp. 784f.), the following parameters and cut–off values were assumed to be acceptable for sufficient model fit: Comparative Fit Index CFI ≥ 0.90, Standardized Root Mean Residual SRMR ≤ 0.08 and Root Mean Square Error of Approximation RMSEA ≤ 0.10. Factor loadings ≥ 0.30 were considered substantial. Because of its dependency on sample size we only calculated Δχ2 and its significance level, without further discussion.

To test for configural, metric and scalar measurement invariance (hypothesis 4) within gender (female vs. male) and age group (<30 vs. ≥30 years), multigroup CFA’s (cf. Weiber and Mühlhaus, 2014, pp. 296f.) were done. Changes in CFI of 0.01, in RMSEA of 0.015 and in SRMSR of 0.02 across all models were considered as equivalent (Vandenberg and Lance, 2000; Chen, 2007). Again, differences in indices other than Δχ^2^ were considered superior to examine differences in model fit (Vandenberg and Lance, 2000, p. 46).

For the pre-post effects (hypothesis 5), *t*-tests for paired samples and Cohen (1988) *d*, respectively *d_*pooled*_*, for mean differences in repeated measurements with one group, together with the corresponding 95% confidence intervals were calculated. The conventions are *d*_*small*_ = 0.20, *d*_*medium*_ = 0.50, *d*_*large*_ = 0.80 (Bortz and Döring, 1995, p. 568), whereby large and medium effects were considered substantial.

## Results

### Item and Scale Analysis

The item and scale means (cf. Table 4) showed no ceiling or floor effects at possible value ranges of 0.00−0.50. The VIL had internal consistencies in terms of Cronbach’s α between 0.72−0.82, McDonnald’s ω ranged from 0.73−0.81, the part-whole correlations were above the cut-off value of 0.30 and below 0.70.

### Construct Validity

Exploratory factor analyses (Maximum Likelihood method with oblimin rotation, eigenvalue > 1.0) yielded five distinct factors. They accounted for 76.67% of the variance. The Kaiser-Meyer-Olkin measure was 0.91, the Bartlett-Test was siginificant (x^2^ = 25 693.16, *df* = 253, *p* < 0.001). There were two cross-loadings with *a* ≥ 0.30 (cf. Table 4).

Higher order exploratory factor analyses with these factor scores (Maximum Likelihood method with varimax rotation, eigenvalue > 1.0) revealed a one-factor solution (eigenvalue factor 1 = 2.63). It accounted for 42.47% of the variance. The Kaiser-Meyer-Olkin measure was 0.76, the Bartlett-Test was significant (x^2^ = 4599.16, *df* = 10, *p* < 0.001). The factor loadings were as follows: *a*_*trust  in  future  perspectives*_ = 0.79, *a*_*goal  clarity*_ = 0.79, *a*_*certainty*_ = 0.69, *a*_*well  being*_ = 0.46 and *a*_*information*_ = 0.44. A. The factor loadings of a forced two-factor solution (Maximum Likelihood method with varimax rotation; KMO = 0.76, x^2^ = 4599.16, *df* = 10, *p* < 0.001, explained variance after rotation = 59.38%, eigenvalue factor 1 = 2.63, eigenvalue factor 2 = 0.89) are shown in Table 5.

**FIGURE 1:**
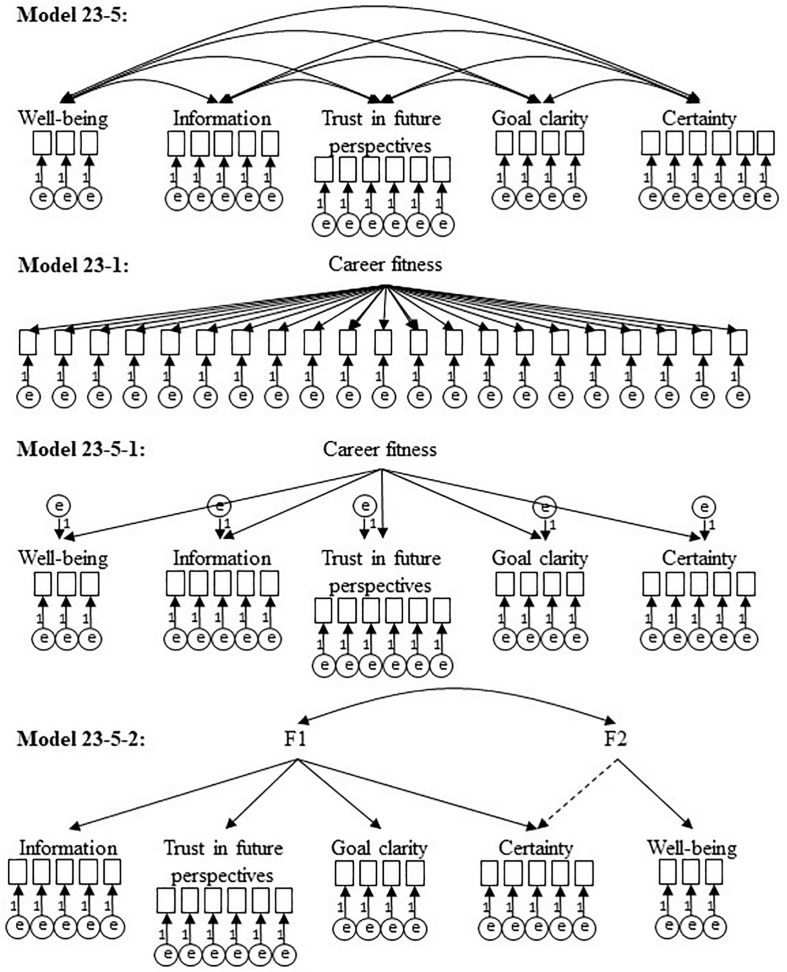
Models tested with CFA.

As a consequence of these preliminary analyses the models shown in Figure 1 were compared by CFA’s: The postulated five-factor model 23-5, two general factor models 23-5-1 and 23-1, respectively and a correlated group factor model 23-5-2.

The results of the model comparisons with CFA are shown in Table 6.

**FIGURE 2:**
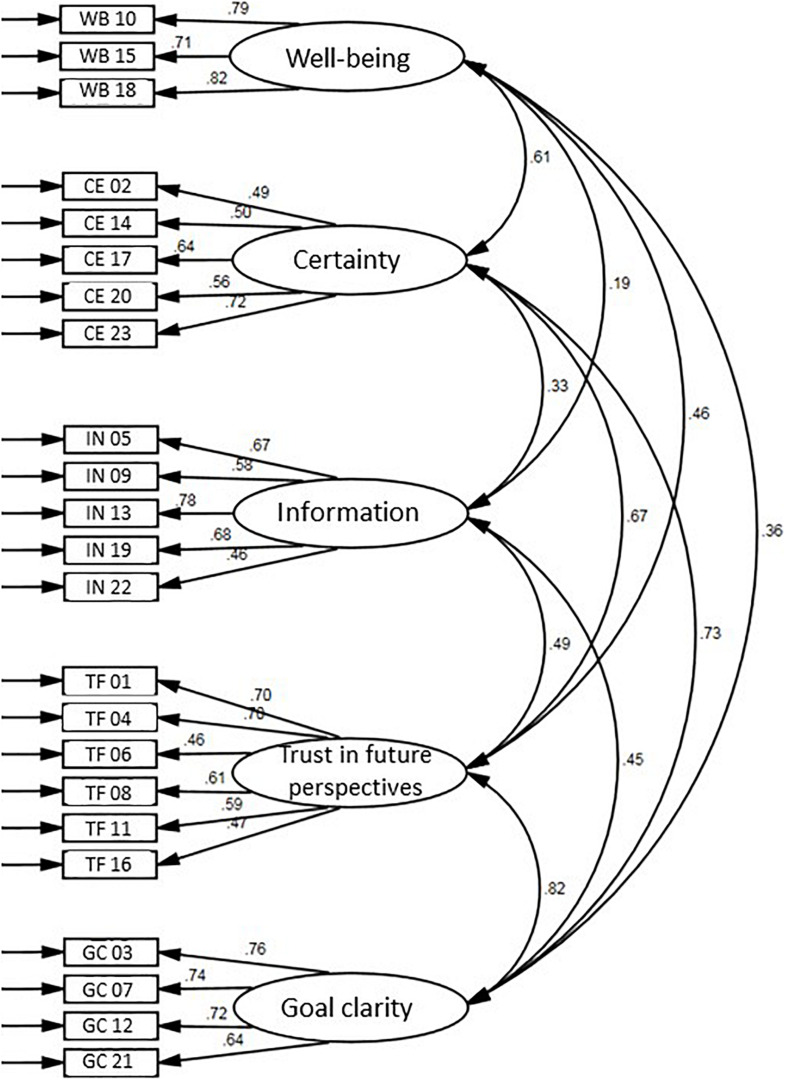
Results of the confirmatory factor analysis (applies to all values: *p* < 0.001).

The parameters of the CFA for the 23-5 model indicated throughout an acceptable fit. The standardized regression weights (cf. Figure 2 and Table 4) ranged between 0.46 ≤ *λ* ≤ 0.82. The consolidation of the highly correlated constructs (*r* ≥ 0.50; cf. Figure 2) *trust in future perspectives* and *goal clarity* led to partly insufficient fit indices and significantly higher *x*^2^-indizes (*x*^2^ = 5689.84, *df* = 224, *p* < 0.001, CFI = 0.786, SRMR = 0.093, RMSEA = 0.086, *CI*_90__(RMSEA)_ = 0.084−0.088, Δ*x*^2^ = 3181.84, Δ*df* = 4, *p* < 0.001). The consolidation of *certainty* and *goal clarity* also led to partly insufficient fit indices and significantly higher *x*^2^-indizes (*x*^2^ = 5890.08, *df* = 224, *p* < 0.001, CFI = 0.778, SRMR = 0.095, RMSEA = 0.087, *CI*_90__(RMSEA)_ = 0.085−0.089, Δ*x*^2^ = 3382.08, Δ*df* = 4, *p* < 0.001), as well as *certainty* and *trust in future perspectives* (*x*^2^ = 5635.33, *df* = 224, *p* < 0.001, CFI = 0.788, SRMR = 0.093, RMSEA = 0.085, *CI*_90__(RMSEA)_ = 0.083−0.087, Δ*x*^2^ = 3127.33, Δ*df* = 4, *p* < 0.001), as well as *certainty* and *well-being* (*x*^2^ = 7071.16, *df* = 224, *p* < 0.001, CFI = 0.732, SRMR = 0.097, RMSEA = 0.096, *CI*_90__(RMSEA)_ = 0.094−0.098, Δ*x*^2^ = 4563.16, Δ*df* = 4, *p* < 0.001).

### Objectives and Hypotheses

This paper is dedicated to the test statistical analysis of the VIL. Five objectives are pursued:

• First, item and scale analyses were performed. It was postulated (hypothesis 1) that the internal consistencies in terms of Cronbach’s α and McDonnald’s ω are above 0.70, as was for Cronbach’s α already reported in Künzli and Zihlmann(2008, pp. 117f.).
• Second, construct validity was checked by means of exploratory factor analysis. It was postulated (hypothesis 2) that the five factors as introduced above can be extracted. In addition, an exploratory factor analysis was conducted with the extracted factor scores to gain an idea of higher order factors.
• Third, construct validity was checked by means of confirmatory factor analysis. It was postulated (hypothesis 3) that a 23-5 factor structure of the VIL scales *well-being*, *certainty*, *information*, *trust in future perspectives* and *goal clarity* can be confirmed against a 23-5-1 or other models derived from the aforementioned higher order exploratory factor analysis.
• Fourth, it was postulated, that there is measurement invariance (hypothesis 4) within gender and two age groups (<30 vs. ≥30 years; since this presumably will lead to two more or less equally sized subsamples and since there ought to be a considerable relocation of the reasons for visiting the career guidance center and a considerable relocation of the last educational qualification as well). Invariance addresses (a) the factor configuration (configural invariance), (b) the factor loadings (metric invariance) and (c) the item intercepts (scalar invariance).
• Fifth, statements on the change sensitivity of the VIL were made on the basis of pre-post comparisons. We assumed (hypothesis 5) that our analyses, compared to those of Künzli and Zihlmann(2008, p. 124), show identical and still considerable effects, i.e., large effects for *goal clarity*, *information* and *certainty*, medium effects for *trust in future perspectives* and *well-being*.

## Materials and Methods

### Design and Setting

The data for the current study was derived from the third project phase (cf. Künzli and Toggweiler, 2014), which was financially supported by the Swiss Federal Commission for Technology and Innovation (KTI) and through funds of the participating cantons. The study was fully checked and approved by the data protection officer of the canton of Zurich. The Federal Act on Research involving Human Beings excluded the study from being subjected to the cantonal Ethics Committee.

Within a naturalistic multicenter study with pre-post design, the VIL was integrated into the standard evaluations of 29 public occupational and career counseling centers from eight cantons with a total of about 260 counselors. The interrogatory process was carefully implemented within the organizational processes of the participating centers. Prior to testing, several cantonal and/or regional information events were held with interested parties and counselors in order to provide information and establish compliance and transparency about the VIL and the upcoming evaluation.

The sample included adult career counseling candidates as described under target groups above. The clients had to undergo one or more individual counseling sessions with a counselor. As far as the counseling concepts, theoretical understanding and procedures of the career counselors were concerned, no restrictions were formulated.

The target group was asked to complete the VIL online three times, between June 2010 and August 2011: immediately after registration for a counseling session (pre-test), after completion (post-test, as soon as no new meeting was dated) and 3 months later (follow-up test). The survey software was linked to the administration software of the counseling centers. This enabled the automated dispatch of invitations across all three polls (1) at case opening with date confirmation by the administration staff, (2) at case closure by the counselors, (3) automatically dispatched 3 months later. Participation in the survey was voluntary and anonymiously. The invitations contained the information that the survey was part of a research project evaluating the progress of career counseling clients. In addition to the information that it would take 5−10 min to complete the questionnaire, a weblink to the questionnaire, an individual user name and a password were provided. The software made the results immediately available to the counselors (via e-mail notification and online access via a personalized link to the survey software) and to the clients (via pop-up window). The use of the pre-poll results for the counseling process was up to the counselors.

### The VIL Questionnaire

The VIL consists of the five scales *well-being*, *information*, *trust in future perspectives*, *goal clarity*, and *certainty* with a total of 23 items, some with negative polarity (cf. Table 4). The items were introduced with the instruction to judge to what extent the individual statements apply. Measurements were made using a continuously adjustable Likert scale with the six verbal anchors *does not apply at all*, *does not apply for the most part*, *tends not to apply*, *tends to apply*, *applies for the most part*, *completely applies*. The range of possible values therefore was from 0.00−0.50. The scale values were calculated by averaging. The definitions of the VIL scales can be found above in the theoretical introductions.

### Sample

During the survey period, 7554 invitations were dispatched for the pre-survey, of which 3439 questionnaires were completed, issuing a response rate of 45.5%. The response rate for the post-survey was 18.1% and 6.3% for the follow-up survey. In total, 957 participant pre-post pairs were available, representing 12.7% of all 7554 clients. The interval between pre- and post-survey was 28 days (median), the first quartile was 15 days. As part of the data screening, all subjects with missing data on age or outside an age range of 17−65 years were excluded. Likewise, 69 clients who were still in compulsory schooling had to be excluded, because they don’t belong to the target group. A further 33 participants were excluded because they consistently showed uniform response behavior (i.e., the same value) across all 23 items of the VIL. Based on highly significant Mahalanobis distances (Tabachnick and Fidell, 1996, p. 94) for the VIL scales, another 21 participants were excluded. The sample was finally composed as shown in Table 3, column total sample.

**TABLE 3:** Description of the sample.

Variable	Total *n* (%)	<30 years *n* (%)	≥30 years *n* (%)
**Gender**			
Female	2159 (65.1)	1205 (66.6)	954 (63.3)
Male	1157 (34.9)	605 (33.4)	552 (36.7)
Age in years (*M* = 30.52, *SD* = 10.42) < 20	458 (13.8)	458 (25.3)	–
20−29	1352 (40.8)	1352 (74.7)	–
≥30	1506 (45.4)	–	1506 (100)
**Last educational qualification**			
Compulsory education	540 (16.3)	464 (25.6)	76 (5.0)
Intermediate year, preparatory year after compulsory school	22 (0.7)	19 (1.0)	3 (0.2)
Vocational education and training (apprenticeship)	1096 (33.1)	575 (31.8)	521 (34.6)
Vocational education and training (apprenticeship) incl. federal vocational baccalaureate	88 (2.7)	81 (4.5)	7 (0.5)
Upper secondary specialized schools, other upper secondary schools	39 (1.2)	27 (1.5)	12 (0.8)
Baccalaureate (i.e., academic high school diploma)	193 (5.8)	158 (8.7)	35 (2.3)
Professional higher education	337 (10.2)	79 (4.4)	258 (17.1)
Universities of applied sciences, universities of teacher education	194 (5.9)	54 (3.0)	140 (9.3)
Universities, Federal Institutes of Technology	259 (7.8)	68 (3.8)	191 (12.7)
Other/unknown	30 (0.9)	11 (0.6)	19 (1.3)
Missing values	518 (15.6)	274 (15.1)	244 (16.2)
**Reason of visiting the career guidance center**			
Vocational or academic choice	429 (12.9)	411 (22.7)	18 (1.2)
Search for an apprenticeship	16 (0.5)	15 (0.8)	1 (0.1)
Problems with education and training (exams, performance, drop-out)	88 (2.7)	83 (4.6)	5 (0.3)
Career planning, vocational reorientation	2001 (60.3)	960 (53.0)	1041 (69.1)
Career re-entry	77 (2.3)	6 (0.3)	71 (4.7)
Unemployment, threat of unemployment	92 (2.8)	22 (1.2)	70 (4.6)
Financial issues	6 (0.2)	2 (0.1)	4 (0.3)
Personal, social and health problems, problems at workplace	56 (1.7)	11 (0.6)	45 (3.0)
Migration, specific topics concerning educational/professional integration	18 (0.5)	10 (0.6)	8 (0.5)
Missing values	533 (16.6)	290 (16.0)	243 (16.1)
Consultations in minutes: *Q*_1_ = 64.74, *Md* = 87.16, *Q*_3_ = 146.73; *Min* = 30, *Max* = 780			
Number of consultations: *Q*_1_ = 1.45, *Md* = 1.45, *Q*_3_ = 2.16; *Min* = 1, *Max* = 13			
Pre-post time interval in days: *Q*_1_ = 15, *Md* = 28, *Q*_3_ = 58			

**N* = 3316. *M*, mean; *SD*, standard deviation; *Md*, median; *Q*_1_, first quartile; *Q*_3_, third quartile.*

**TABLE 4:** Item and scale analyses as well as factor loadings of the EFA and standardized regression weights of the CFA.

		*M*	*SD*	*r_*it*_*	α	ω	*d_*pooled*_ (*N* = 957)*	*d'*(*N* = 539–559)	1	2	3	4	5	λ
**Well–being**		0.23	0.11		0.82	0.81	0.61***	0.70						
	(10) At the time being I feel very balanced.	0.22	0.12	0.68		[0.80, 0.82]	[0.51, 0.69]		0.05	**−0.80**	0.03	−0.08	0.00	0.79
	(15) *At the time being I feel pretty tense.*	0.23	0.14	0.63					−0.16	**−0.73**	−0.02	0.10	−0.03	0.71
	(18) At the time being I feel very comfortable.	0.26	0.13	0.69					0.10	−**0.80**	0.00	−0.03	0.04	0.82
**Information**		0.23	0.08		0.77	0.77	1.49***	1.73						
	(5) I know my education and training opportunities.	0.22	0.12	0.58		[0.76, 0.78]	[1.36, 1.56]		−0.01	−0.05	**0.69**	−0.02	0.03	0.67
	(9) *I know too little about all the possibilities (professions*, *trainings*, *etc*.).	0.20	0.12	0.51					−0.17	0.04	**0.61**	0.15	−0.08	0.58
	(13) I’m well informed about the career opportunities that interest me.	0.21	0.11	0.64					0.03	0.00	**0.75**	−0.16	−0.16	0.78
	(19) I have clear enough information about the possibilities that are appropriate for me.	0.19	0.11	0.57					0.13	0.01	**0.63**	−0.07	−0.03	0.68
	(22) I know how to get key information.	0.30	0.11	0.41					0.14	−0.03	**0.43**	0.09	0.15	0.46
**Trust in future perspectives**		0.29	0.08		0.76	0.76	0.87***	0.65						
	(1) I can see opportunities in which I can apply my interests and abilities.	0.29	0.10	0.59		[0.75, 0.77]	[0.80, 0.99]		**0.56**	−0.04	0.11	−0.05	−0.20	0.70
	(4) I see (professional) opportunities that suit me.	0.30	0.12	0.56					**0.55**	0.01	0.07	−0.03	−0.26	0.70
	(6) I am aware of what kind of environment I need so that I can best develop my strengths.	0.30	0.12	0.44					**0.47**	0.02	0.04	0.03	−0.01	0.46
	(8) I’m convinced that I’m on the right track.	0.26	0.12	0.51					**0.42**	−0.10	0.01	0.30	0.00	0.61
	(11) I see promising tracks into my future.	0.28	0.11	0.50					**0.53**	−0.12	0.03	0.13	0.06	0.59
	(16) I can clearly distinguish between solutions that are suitable for me and solutions that are not.	0.31	0.11	0.43					**0.37**	−0.05	−0.01	0.12	−0.07	0.47
**Goal clarity**		0.25	0.11		0.81	0.81	0.94***	1.22						
	(3) *Right now*, *I have no idea of what I want.*	0.26	0.15	0.67		[0.80, 0.82]	[0.81, 1.00]		0.15	−0.04	−0.05	0.17	−**0.61**	0.77
	(7) *My career goals are unclear.*	0.25	0.13	0.64					0.06	−0.05	0.10	0.22	−**0.54**	0.74
	(12) I have clear ideas of what I want.	0.25	0.13	0.62					0.40	−0.03	0.02	−0.04	−**0.49**	0.73
	(21) *I only have vague ideas as to which direction I might move in.*	0.23	0.13	0.57					0.05	−0.02	0.12	0.10	−**0.53**	0.64
**Certainty**		0.22	0.09		0.72	0.73	1.04***	1.28						
	(2) *I wonder whether I’m heading in the right direction.*	0.20	0.12	0.43		[0.71, 0.74]	[0.99, 1.18]		0.00	0.00	−0.04	**0.52**	−0.06	0.49
	(14) *I hesitate in implementing a plan.*	0.27	0.14	0.43					0.12	−0.03	−0.03	**0.45**	0.00	0.50
	(17) *I go around in circles searching for (professional) solutions.*	0.22	0.13	0.50					0.03	−0.10	0.03	**0.44**	−0.20	0.64
	(20) *I doubt that the possibilities that I am thinking about are really possible for me.*	0.22	0.13	0.51					0.01	0.01	0.09	**0.58**	0.00	0.56
	(23) *I’m confused about my future.*	0.19	0.14	0.55					0.02	−0.25	0.07	**0.49**	−0.10	0.72

Inverted items are printed in italics. Original German wording c.f. Table 1. *N* = 3316. ***p ≤ 0.001 (*t*–Test for dependent samples); *M*, mean; *SD*, standard deviation; *r*_*it*_, part–whole correlation; α, Cronbach’s Alpha; ω, McDonnald’s ω with 95% confidence interval; *d*_*pooled*_, effect size (Morris and DeShon, 2002) for repeat measurements with one group; *d_95__%_*, 95% confidence interval of effect size *d*_*pooled*_; *d’*, effect size for mean differences (Cohen, 1988) of two equal groups in the study by Künzli and Zihlmann(2008, p. 124); 1−5, factors 1 to 5 resulting from the EFA, main loadings are printed in bold, cross loadings are underlined; KMO = 0.91, Bartlett–Test Chi–Quadrat = 25693.16, *df* = 253, *p* < 0.001, explained variance after rotation = 76.65%; λ, regression weights of the CFA.

**TABLE 5:** Forced two-factors solution of the higher order EFA.

	1	2
Goal clarity	0.83	
Trust in future perspectives	0.74	
Certainty	0.60	0.34
Information	0.43	
Well-being		0.98

**N* = 3316. KMO = 0.91, Bartlett-Test Chi-Quadrat = 4599.16, *df* = 10, *p* < 0.001, explained variance = 59.38%.*

The fit indices for the 23-1 general factor models (cf. Table 6) did not reach acceptable values by far, whereas the 23-5-1 model was fairly good, but differed significantly in RMSEA (cf. their confidence intervals) from the 23-5 model. The CFI of the correlated group factor model 23-5-2 was also substantially below the quality of the 23-5 model (cf. CFI, RMSEA and their confidence intervals, respectively). All freely estimated factor loadings were statistically significant (*p* < 0.001).

**TABLE 6:** Results of CFA, model comparisons and measurement invariance.

Model	*x*^2^	*df*	CFI	SRMR	RMSEA	*CI*_90__(RMSEA)_	Δ*x*^2^/Δ*_*df*_*
23-5	2508.00***	220	0.910	0.044	0.056	0.054, 0.058	
23-1	9327.66***	230	0.643	0.091	0.109	0.107, 0.111	6819.66***/10
23-5-1	2867.49***	225	0.896	0.051	0.060	0.058, 0.064	359.49***/5
23-5-2	6258.23***	228	0.764	0.075	0.089	0.087, 0.091	3750.23***/8
Multigroup 23-5 CFA for gender (*n*_*f*_ = 2161, *n*_*m*_ = 1155)							
Configural	2767.64***	440	0.909	0.045	0.040	0.039, 0.041	
Metric	2795.28***	458	0.909	0.045	0.039	0.038, 0.041	27.64/18
Scalar	2915.10***	481	0.905	0.045	0.039	0.038, 0.040	119.82***/23
Multigroup 23-5 CFA for age group (*n*_<__30 Jahre_ = 1810, *n*_≥__30 Jahre_ = 1506)							
Configural	2803.28***	440	0.908	0.049	0.040	0.039, 0.042	
Metric	2872.06***	458	0.906	0.050	0.040	0.038, 0.041	68.78***/18
Scalar	3209.47***	481	0.894	0.050	0.041	0.040, 0.043	337.41***/23

*N* = 3316. ***p < 0.001. CFI, comparative fit index; SRMR, standardized root mean-squared residual; RMSEA, root mean square error of approximation; *CI*, confidence interval.

As reported in Table 6, the configural, metric, and scalar invariance models for gender (female vs. male) evidenced full equivalence of the VIL. Concerning age groups (<30 vs. ≥30 years, for group characteristics cf. Table 3), there is configural and metric invariance. With respect to Chen (2007), scalar invariance was missed by 0.004 in the CFI value, whereas the differences in SRMS and RMSEA indicated scalar equivalence of the VIL. All freely estimated factor loadings were statistically significant (*p* < 0.001).

### Counseling-Correlated Change Effects

**FIGURE 3:**
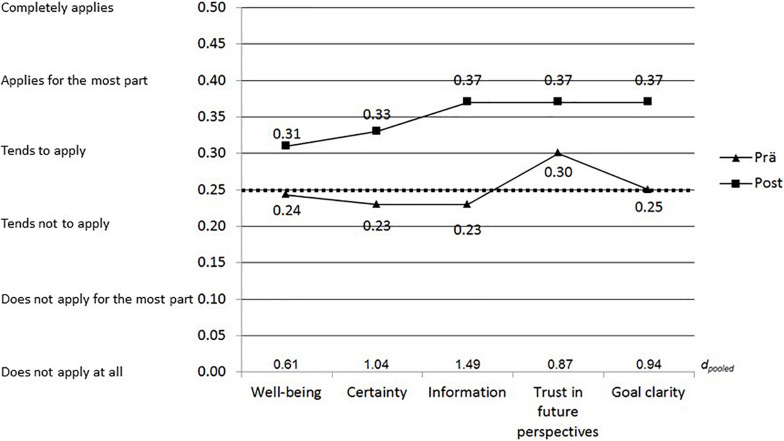
Pre-post-effects (*N* = 957).

The pre-post effects *d*_*pooled*_ (cf. Figure 3 and Table 4) for mean differences in repeated measurement for one group (Morris and DeShon, 2002) are medium for *well-being* (*d*_*pooled*_ = 0.61) and large for *trust in future perspectives* (*d*_*pooled*_ = 0.87), *goal clarity* (*d*_*pooled*_ = 0.94), *certainty* (*d*_*pooled*_ = 1.04), and *information* (*d*_*pooled*_ = 1.49). The effects *d* (Cohen, 1988) calculated by Künzli and Zihlmann(2008, p. 124) are outside the 95% confidence intervals of *d*_*pooled*_ throughout. Due to a response rate of only 6.3% in the follow-up survey no such analyses were made.

## Discussion

The aim of this study was to analyze and describe the psychometric properties of the VIL. Therefore, item and scale analyses, confirmatory factor analyses and a pre-post comparison were performed. The exclusions of some few outliers in the process of data cleaning proved to be insignificant with regard to the results.

In terms of the target group, a large sample of subjects older than 20 years focusing on career planning and vocational reorientation, as well as on occupational and academic choices, collected within a naturalistic multicenter study with pre-post design, it is appropriate to draw conclusions about the psychometric properties of the VIL. The widespread distribution of the educational qualifications is also advantageous for examining the VIL as a broadly applicable screening instrument.

As far as the internal consistencies of the scales are concerned, hypothesis 1 (all Cronbach’s α and McDonnald’s ω are above 0.70) could be accepted. All dimensions measured with appropriate reliability.

The five dimensions of the 23-5 model could be confirmed by exploratory (hypothesis 2) and confirmatory (hypothesis 3) factor analysis with an acceptable model fit. Alternative models (two general factor models 23-5-1 and 23-1, a correlated group factor model 23-5-2, several consolidation of highly correlated latent variables) only achieved insufficient or significantly lowered model fits. Therefore, the dimensions of the VIL theoretically and practically must be treated as separate and equivalent constructs.

Concerning configural, metric and scalar measurement invariance (hypothesis 4), gender evidenced as fully equivalent. So VIL results of male and female samples may be generalized and are fully comparable. On the other hand, age group undoubtedly evidenced configural and metric invariance, but slightly missed scalar invariance by 0.004 (at an absolute difference of −0.014) in CFI. In this context Vandenberg and Lance(2000, p. 46) confirm that “changes in CFI of −0.01 or less indicate that the invariance hypothesis should not be rejected, but when the differences lie between −0.01 and −0.02, the researcher should be suspicious that differences exist. Definite differences between models exist when the change in CFI is greater than −0.02”. So one should not overestimate the observed difference of −0.014 in CFI, since simultaneously both the SRMR and RMSEA confirm scalar equivalence. So far, there is no overwhelming evidence that differences between different age groups of clients must not be calculated, as the measurement scales are not at all fundamentally different across age groups. To further qualify this situation, one must consider some correlates of age group (cf. Table 3), namely differences, which constitute these two groups in a differential sense. Along the axis of educational qualification, the following predictors appear to be highly discriminant: Compulsory education, baccalaureate (i.e., academic high school diploma), professional higher education, and universities. In addition, and along the axis of the specified reasons for visiting the career guidance center, the two groups may be differentiated by vocational or academic choice, and career planning/vocational reorientation. Taken together, the two age groups may be distinguished by pronounced bundles of qualifications and career concerns, undoubtedly bad prerequisites for unbiased values of measurement invariance.

Hence, measurement equivalence for gender must be accepted and there is good evidence to suppose measurement invariance concerning age as well. This means that the current data does not only allow for constructions of nomolocical networks with the VIL (as it is the case with configural and metric invariance), but also in good conscience for absolute comparisons of scale scores due to similar scale metrics.

The intercorrelations of the five latent variables ranged between 0.19 and 0.82, with only *well-being* and *information* being small, i.e., below 0.30. The other intercorrelations showed medium to large effect sizes, which indicates, for the time being, unexplained but significant dependencies and/or interactions. *Well-being* was most markedly associated with *certainty*, in other words, when a client’s decision and conviction about further career steps emerged. On the other hand, *well-being* was least affected by *information*, i.e., the rational aspect of information processing. Furthermore, *certainty* regarding a decision turned out to be important. It went hand in hand with *trust in future perspectives* and *goal clarity*. These results allow the conclusion that it is promising to carry out cross-lagged studies in order to clarify these relations more precisely.

Addressing the pre-post comparisons (hypothesis 5: Identical and still considerable change effects compared to the analyses of Künzli and Zihlmann, 2008, p. 124), there occured consistent discrepancies. The effect sizes of *well-being*, *certainty*, *information* and *goal clarity* significantly decreased, while that of *trust in future perspectives* significantly increased. Nonetheless, the effects remained considerable, i.e., medium to large, but were not identical to those of the previous study. Since it is of crucial importance to use short instruments in the given context, some loss is acceptable. The *information* scale once again played the most important role, followed by *certainty*. It is unclear whether the reported differences were due to the adapted instrument and/or to differences in the samples. In addition, the issue of the response rate is critical: a 45.5% response-rate in the pre-survey was remarkably lower than the 81.1% of Künzli and Zihlmann(2008, pp. 116f.). The response rate of 12.7% for pre-post pairs was also remarkably lower than the 35.5% reported by Künzli and Zihlmann(2008, p. 123).

Based on 28 individual interviews lasting around two hours with participating career counselors, Künzli and Toggweiler(2014, p. 13) stated that the VIL was well received. It was used in many ways. Some of the career counselors used the VIL primarily as an evaluation tool, meaning that they were particularly interested in the post-surveys. Others saw the main benefit in the pre-survey, as it provided an opportunity to anticipate and to plan their counseling process (Künzli and Toggweiler, 2014, p. 13). However, the greatest benefit to the counselors was the feedback they received on their work itself, which enabled them to improve their counseling quality. In addition, they attributed a motivating effect to the feedback, in that the progress of those seeking advice was depicted visually and numerically. Moreover, most clients reported no problem filling out the VIL (Künzli and Toggweiler, 2014, p. 13).

Finally, there needs to be some discussion of various interfering variables that limited the internal validity of the present study. It is possible that changes in the VIL scales had occurred even before the first consultation, due to the attention given to the topic, and the fact that an important step had been taken to tackle it. It is also conceivable that other counseling and, in particular, information services have been used, which of course distorted the “effect” of the ongoing counseling sessions. Furthermore, there were various process variables that were supposed to also have been influential, e.g., family support, availability of supportive friends, availability of information material and services. For this reason, it is important (a) to refer to “counseling-related changes” and not to “counseling effects,” (b) to adapt the research questions and the design of the studies accordingly.

Our conclusions are as follows: As the sample size is huge, we can rely on the assumption of good psychometric properties of the five VIL dimensions. Cronbach’s Alphas and McDonald’s Omegas are sufficient, factorial validity is obvious. Measurement invariance of the VIL has been proved for gender and is supposed for age as well.

Concerning the limitations of the study, what needs to be tackled next is to analyze the nomological network, i.e., analyze the convergent and discriminant validity of the VIL (cf. Table 2) with the *Career Adaptability Scales* (Savickas and Porfeli, 2012; Johnston et al., 2013) or with the *Career Resources Questionnaire* by Hirschi et al. (2018). Furthermore, the interrelations within the VIL must be described using structural equation models. In addition, other constructs that indicate successful counseling outcomes must be included, such as *Career Decision-Making* (Stangl and Seifert, 1986; Gati et al., 1996; Joerin et al., 2004; Ebner et al., 2016) or the *Future Work Self* (Ferrari et al., 2012; Strauss et al., 2012), as well as *Career Self-Efficacy* (Hirschi et al., 2018).

## Data Availability Statement

The datasets presented in this article are available upon request. Requests should be directed to stephan.toggweiler@zhaw.ch.

## Ethics Statement

Ethical review and approval was not required for the study on human participants in accordance with the local legislation and institutional requirements. Written informed consent from the participants’ legal guardian/next of kin was not required to participate in this study in accordance with the national legislation and the institutional requirements.

## Author Contributions

HK contributed to theory and construction of the original instrument. ST contributed to upgrade the original instrument and theory and analyses. Both authors contributed to the article and approved the submitted version.

## Conflict of Interest

The authors declare that the research was conducted in the absence of any commercial or financial relationships that could be construed as a potential conflict of interest.
